# Multisite *de novo* mutations in human offspring after paternal exposure to ionizing radiation

**DOI:** 10.1038/s41598-018-33066-x

**Published:** 2018-10-02

**Authors:** Manuel Holtgrewe, Alexej Knaus, Gabriele Hildebrand, Jean-Tori Pantel, Miguel Rodriguez de los Santos, Kornelia Neveling, Jakob Goldmann, Max Schubach, Marten Jäger, Marie Coutelier, Stefan Mundlos, Dieter Beule, Karl Sperling, Peter Michael Krawitz

**Affiliations:** 1 Berlin Institute of Health (BIH), Core Unit Bioinformatics, Berlin, 10178 Germany; 2 Institute for Genomic Statistics and Bioinformatics, Rheinische Friedrich-Wilhelms Universität, Bonn, 53127 Germany; 3 Charité – Universitätsmedizin Berlin, Institute of Medical and Human Genetics, 13353 Berlin, Germany; 4 Charité – Universitätsmedizin Berlin, Berlin, 10117 Germany; 5 Department for Human Genetics, Radboud University, Nijmegen, 6525 Netherlands; 6 Berlin Institute of Health (BIH), JRG Computational Genome Biology, 10178 Berlin, Germany; 7 Max Delbrück Center for Molecuar Medicine, 13125 Berlin, Germany

## Abstract

A genome-wide evaluation of the effects of ionizing radiation on mutation induction in the mouse germline has identified multisite *de novo* mutations (MSDNs) as marker for previous exposure. Here we present the results of a small pilot study of whole genome sequencing in offspring of soldiers who served in radar units on weapon systems that were emitting high-frequency radiation. We found cases of exceptionally high MSDN rates as well as an increased mean in our cohort: While a MSDN mutation is detected in average in 1 out of 5 offspring of unexposed controls, we observed 12 MSDNs in altogether 18 offspring, including a family with 6 MSDNs in 3 offspring. Moreover, we found two translocations, also resulting from neighboring mutations. Our findings indicate that MSDNs might be suited in principle for the assessment of DNA damage from ionizing radiation also in humans. However, as exact person-related dose values in risk groups are usually not available, the interpretation of MSDNs in single families would benefit from larger molecular epidemiologic studies on this new biomarker.

## Introduction

**Figure 1:**
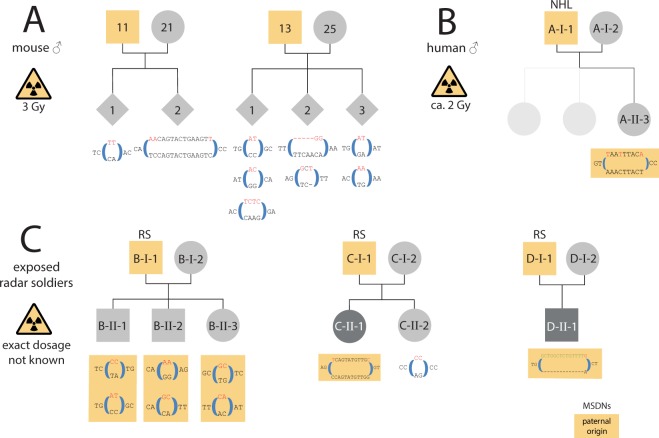
Multisite *de novo* mutations (MSDNs): (**A**) Offspring of male mice that were irradiated with 3 Gy showed a significantly increased rate of MSDNs^1^. (**B**) The daughter of a Non Hodgkin Lymphoma (NHL) patient that was fathered after irradiation therapy had a MSDN of paternal origin. (**C**) Offspring of some radar soldiers (RS) that were potentially exposed to high frequency radiation during maintenance work, showed high rates of MSDNs. Depicted are families with at least one MSDN in at least one offspring. All 7 MSDNs for which the parental origin could be determined, are from the paternal germline (yellow shaded). Furthermore, a balanced (C-II-1) as well as an unbalanced translocation (D-II-1) could be identified that also originate from the paternal germline (see Fig. 2).

Improving our understanding of the effects of ionizing radiations on the human genome was one of the main motivations for the Department of Energy of the United States to initiate the Human Genome Project^1^. As already noted 30 years ago, the most straightforward approach to looking for mutations is by determining the sequence of every nucleotide in a child´s genome and then comparing this with the DNA sequence of the child’s parents^2^. Today, high-throughput sequencing technology enables us to analyze sequence variants at a genome-wide level and *de novo* rates for different mutational classes as well as their influencing factors have been investigated in several population-scale studies^3–6^. However, the consequences of ionizing radiation for offspring of exposed humans have not yet been worked up with this technology and molecular epidemiological data on risk cohorts only exists on mini-satellites and chromosomal aneuploidies^7–9^. While the results of these studies suggest a higher vulnerability to ionizing radiation during germ line maturation, a major limitation of these biomarkers is that the number of mutational events that can be measured per offspring is restricted. A recent study in mice pointed to multisite *de novo* mutations (MSDNs) as a potential biomarker that can be measured genome-wide and that might therefore be more sensitive to assess damage from high frequency radiation in single families^10,11^: MSDNs are defined as two or more lesions within 20 bp and in offspring of male mice that were irradiated with 3 Gy, MSDNs occurred at a rate that is nine times higher than in controls (Fig. 1A)^12^.

**Figure 2:**
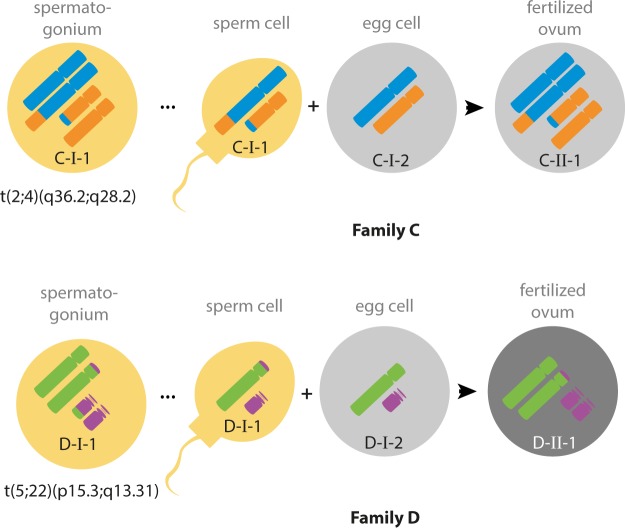
*de novo* chromosomal rearrangements in the paternal germline. Cri-du-chat syndrome observed in D-II-1, is explained be the a *de novo* deletion of 5p, the molecular causes of the severe limb malformation in B-II-1 is yet under investigation (Supplemental Figs 7 and 8).

To our knowledge, we are the first to analyze MSDN rates in a cohort of human families with a potential exposure to high doses of ionizing radiation (IR). We analyzed one trio where the father was treated for Non Hodgkin lymphoma involving radiotherapy of the inguinal lymph nodes. Although the testes had been shielded during fractionated irradiation, the total dosage on the germline was estimated to be up to 2 Gy. Furthermore, we recruited 12 families with altogether 18 offspring, in which the fathers served as radar soldiers in the German military in the late 1950s to early 1980s. It was later found that some of the technical equipment they operated, had not been optimally shielded and leaked high frequency radiation^13^. Weapon systems such as the HAWK, NIKE, SGR 103, and P15 exposed individuals to local doses of 150 mSv/h and even up400 mSv/h (SGR-103). Thus, during their entire duty at the radar units, these soldiers might have been exposed to high doses of IR and even though no exact doses can be determined in retrospective, different kinds of cancers have been recognized as occupational illnesses for this professional group.

## Methods

### Study cohort

In total, *de novo* mutations were analyzed in 18 offspring of radar soldiers with a potential exposure to ionizing radiation (IR) and 1 offspring of a Non-Hodgkin Lymphoma (NHL) patient who was subjected to radiotherapy. All experiments were carried out in accordance with the relevant guidelines and regulations. The ethics board of Charité-Universitätsmedizin Berlin approved this study, and informed consent was obtained from all participants. In addition, we analyzed the genomes of 28 offspring of parents without known exposure to IR. In the following, we also refer to the radar soldier cohort as “exposed” and their offspring as “cases” and the unexposed cohort as “controls”. In Fig. 1, we only depict pedigrees and MSDNs of four families that are discussed in more detail in the results section: Family A because of the good estimate for the dose, family B because of the extraordinary high rate of tandem *de novo mutations*, and families C and D because of the additional occurrence of chromosomal rearrangements. The additional three MSDNs of the exposed cohort that are not visualized in Fig. 1, as well as the MSDNs of the control cohort are listed in Table S3.

### Sequencing and bioinformatics

Genomic DNA was obtained from blood or saliva samples and libraries were constructed according to the TruSeq PCR free or nano protocol depending on the amount of available DNA. After passing quality control, DNA fragments with an insert size between 340–570 bp were subjected to sequencing on a HiSeq X Ten platform with 150 bp paired-end protocol until minimum mean coverage of 30x per sample was reached. The sequence reads were aligned using BWA-MEM, duplicates were masked using Samblaster, and the resulting SAM files were converted to BAM and sorted using Samtools^14–16^. SNVs and small indels were called using the UnifiedGenotyper (after realignment) and the HaplotypeCaller tools from GATK with default settings^17^.

SNVs and small indels were filtered to a high-confidence *de novo* candidate call sets in a fashion similar to the one described by Besenbacher *et al*.^18^ First, variants were filtered to those showing a *de novo* genotype pattern: heterozygous alternative in index and homozygous reference in the parents. Second, variants were excluded falling into the UCSC “simpleRepeat” or “repeat_masker” track and also those having a value below 1 in the UCSC track “wgEncodeCrgMapabilityAlign36mer”. Third, a minimum genotype quality of 50 was required, a coverage between 10 and 120 reads, and a fraction of alternative reads at the variant site between 0.2 and 0.8. Finally, we applied filters similar to Wong *et al*. to remove potential false positives caused by mapping errors^5^. For MSDNs that were subjected to Sanger validation and PacBio phasing, we dropped the criteria that no additional *de novo* calls within 1000 bp have been made, but we required detection by both calling tools, UnifiedGenotyper and HaplotypeCaller. The scripts that we used for filtering are also available upon request.

Phasing was performed on the high-confidence *de novo* SNV and small indel sets using the PhaseByTransmission and ReadBackedPhasing tools of GATK and then extracted using a similar approach as in Wong *et al*.^5^. As ReadBackedPhasing explores a number of haplotypes that is exponentially growing with the number of variants which leads to too high running times, we excluded regions with too many variants. For this, we split the genomes into windows of length 10kbp and ignored them if they contained more than 100 variants. Of the *de novo* SNVs 34% could be phased, which is a ratio consistent with Wong *et al*.^5^.

As read-backed phasing with Illumina sequences is only possible over short distances, we generated long range PCR products for all MSDNs that could not be phased with Illumina data in the exposed cohort. PacBio reads were aligned with NGMLR version 0.2.2 and then postprocessed by the “bamChain” program provided by Pacific Biosciences^19^. Finally, the aligned reads were sorted using Samtools. Figure S5 shows an example of phasing a MSDN event in index B-II-1 using PacBio data. Two PacBio reads connect the allele with the MSDN in the child with the closest informative SNP in the father in a distance of roughly 4 kb.

We used IGV for viewing both Illumina and PacBio data and screened the MSDN candidates for nearby informative SNPs that could be used for assigning the *de novo* to maternal or paternal origin^20^.

All MSDNs and phase informative SNPs in a distance below 500 bp were confirmed by Sanger sequencing of genomic DNA. Where the distance between the MSDN and the phase informative SNP exceeded 500 bp we subcloned the maternal and paternal alleles in the pT7T3U19 plasmid and sequenced the clones.

## Results

In total, we subjected 19 offspring of potentially exposed fathers to whole genome sequencing (WGS) according to protocols that are similar to the large population studies that were recently conducted to assess the parental age influence on germline *de novo* mutations^3–6^. We measured *de novo* mutation rates for single nucleotide variants (SNV) that are comparable to these studies and could also confirm the paternal and maternal age effect in our cohort (Supplemental Figs 1, 2 and 3). After accounting for age, we could not find significant differences in the total number of DNMs in the exposed and unexposed cohort.

In addition to isolated *de novo* SNVs, we also detected thirteen MSDNs in eleven offspring of seven fathers in our pilot study (Table 1, Fig. 1B,C). The *de novo* status has been confirmed for all MSDNs by Sanger sequencing and for eleven MSDNs we were also able to determine the parental origin by read-backed phasing: The MSDN in the NHL offspring as well as all 11 phased MSDNs in offspring of radar soldiers occurred in the paternal germline. 5 MSDNs were phased with informative heterozygous SNPs in more than 4 kb distance by long read sequencing data from the PacBio platform (see Figs S4 and S5). We also applied the same methods of MSDN detection to a control trio cohort of 28 offspring with limb malformations and could confirm only five MSDNs. Thus, the MSDN mutation rate per offspring in the potentially exposed cohort was 0.68 (13/19) compared to 0.18 (5/28) in the control.

**Table 1:** *DNMs* in exposed and unexposed cases and parental origin if ascertainable.

Cohort	Subgroup	n	DNMs	Paternal	Maternal	NA	MSDN	P	M	NA
exposed	NHL	1	74	209	45	582	1	1	0	0
	Radar	18	762				12	11	0	1
control	Limb	28	1639	393	167	1079	5	3	0	2

Interestingly, there is a subclass of MSDNs with immediately adjoining nucleotide exchanges, also referred to as tandem mutations. We found two tandem mutations in the control cohort compared to a total of 1,639 *de novo* SNV calls (Table 1). This proportion is slightly smaller than the values of 0.3% and 0.4% that have previously been reported^21,22^. In contrast, we found seven tandem mutations in the test cohort, six of them in three offspring of a single family (Fig. 1). Thus, in this family the relative tandem substitution rate is extraordinarily high, whereas the total number of *de novo* SNVs (B-II-1: 41, B-II-2: 44, B-II-3: 46) fits to the expectations for the parental ages (Supplemental Fig. 2). This ratio is astonishingly close to the observations of five tandems and 99 SNVs in Adewoye *et al*.^11^. It is therefore an intriguing question whether not the total number of *de novo* SNVs is increased in human offspring after exposure to irradiation but only the fraction that is densely clustered and also more difficult to repair^23,24^. *In silico* simulations do not only suggest that the number of lesions in a cluster is correlated positively with the linear energy transfer of the ionizing radiation, it is also a mutational class that might be especially difficult to repair when occurring post-meiotically^24,25^.

Moreover, two offspring in families that also exhibit MSDNs, have *de novo* translocations. In C-II-1 the balanced translocation t(2;4)(q36.2;q28.2) was identified after multiple miscarriages and in D-II-1 cri-du-chat syndrome was molecularly confirmed by a deletion-duplication due to t(5;22)(p15.3;q13.31) (Fig. 2). In the WGS data, we found read-pairs that map to both chromosomes and that allowed us to identify the exact breakpoints. With informative SNPs on read-pairs that span the breakpoint, we were able to show that both translocations must have occurred in the paternal germlines (Figs S5 and S6).

Such translocations result from two closely neighboring DSBs on different chromosomes that are inappropriately repaired by non-homologues end joining. The “spontaneous” incidence of such reciprocal translocations is below one in a thousand pregnancies^26,27^.

## Conclusion

We analyzed the MSDNs in a cohort of offspring that were conceived while their fathers were at risk of exposure to high frequency radiation and observed rates that were higher than in a control cohort that was analyzed with the same protocols. Personal communications with the Inova study group confirmed that the event rate for paternal MSDNs in the general population is estimated to be 0.23 and the Poisson probability to observe 11 or more paternal MSDNs in 18 offspring with this rate parameter is below 0.005^28^. The results of our small pilot study are certainly encouraging to analyze MSDN rates in larger case cohorts and we started recruiting for a follow up study (https://www.igsb.uni-bonn.de/en/research/radar). With highly accurate long-read sequencing technology it is especially intriguing to hypothesize that radiation induced mutations in mini-satellites in combinations MSDNs in offspring genomes might serve as a dosimeter for the cumulative radiation exposure of their parents.

## Electronic supplementary material

Supplemental Material
