# Supplementary material for: Multisite de novo mutations in human offspring after paternal exposure to ionizing radiation
Source: Sci Rep. 2018 Oct 2;8:14611. doi: 10.1038/s41598-018-33066-x (PMC6168503; doi:10.1038/s41598-018-33066-x)
Supplement: Supplementary file 1 — Supplemental Material [file 41598_2018_33066_MOESM1_ESM.docx]

**Supplemental Material**

**Multisite *de novo* mutations in human offspring after paternal exposure to ionizing radiation**

Manuel Holtgrewe, Alexej Knaus, Gabriele Hildebrand, Jean-Tori Pantel, Miguel Rodriguez de los Santos, Kornelia Neveling, Jakob Goldmann, Max Schubach, Marten Jäger, Marie Coutelier, Stefan Mundlos, Dieter Beule, Karl Sperling, Peter Krawitz


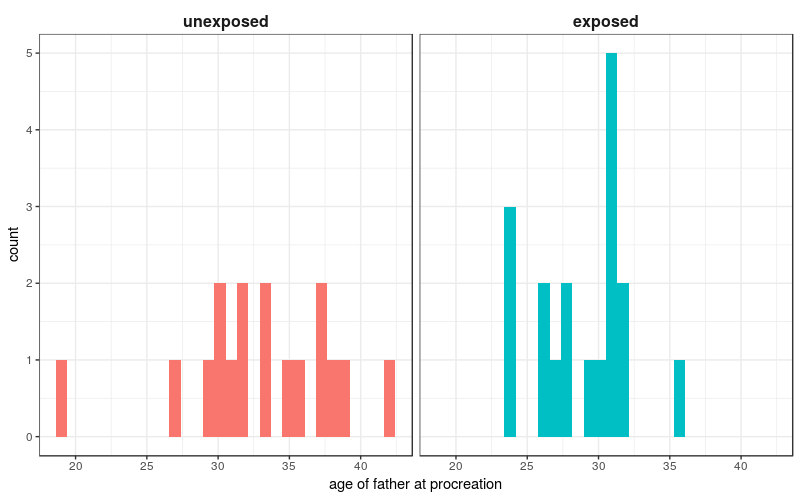
**Figure S1:** Distribution of age of fathers at time of procreation in case and control cohort. For 11 of the control offspring we could not determine the age of at the parents at the age of procreation and therefore excluded these individuals from the following analysis of mutation rates and parental age effects.


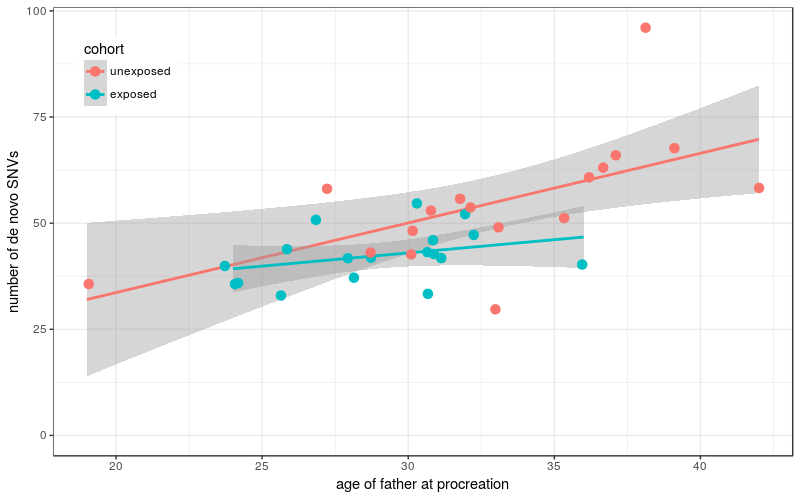
**Figure S2:** Number of *de novo* SNVs positively correlates with the age of fathers at procreation for the case and control cohort. In The lines were drawn with the “geom_smooth(method=lm)” function of R package ggplot2 which uses a linear model. The shaded area indicates the standard error.


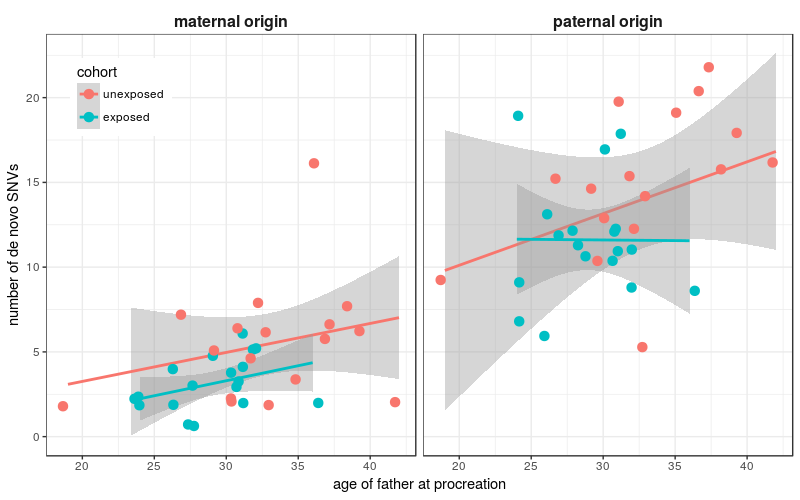
**Figure S3:** Number of *de novo* SNVs for the age of father at procreation for the phased SNVs (cf. Figure S2).

## Single Nucleotide Variants (SNV) de novo Rate Age Effects

We chose the following linear model for testing the influence of age *a* and exposure δ on the number of *de novo* SNVs γ.

γ = β_0_ + δ ∙ β_1_ + *a* ∙ β_2_ + δ ∙ *a* ∙ β_3_ (1)

The model was fit with the R function “lm” and the model parameters were estimated as show in Table S1.

| **Parameters** | **Estimate** | **Std. Error** | ***t* value** | **Pr(>\|*t*\|)** |
| --- | --- | --- | --- | --- |
| β_0_ | 0.8431 | 14.8583 | 0.057 | 0.955114 |
| β_1_ | 23.4784 | 25.1817 | 0.932 | 0.358355 |
| β_2_ | 1.6405 | 0.4455 | 3.682 | ****0.000877** |
| β_3_ | 1.0182 | 0.8282 | -1.229 | 0.228152 |

**Table S1: Parameters of model fit for unphased SNV *de novo* count.**

While the age parameter β_2_ has a statistically significant effect at a confidence level of 0.001, neither the exposure parameters β_1_ nor β_3_ have any significant effect. The adjusted R-squared was 0.487.

## Phasing of MSDNs

As the number of MSDNs was relatively low, we manually inspected the loci around the MSDN and phased them based on the read signal. All MSDNs and phase informative SNPs in a distance below 500bp were confirmed by Sanger sequencing of genomic DNA. Where the distance between the MSDN and the phase informative SNP exceeded 500bp we subcloned the maternal and paternal alleles in the pT7T3U19 plasmid and sequenced the clones (example: MSDN cluster 4). For one trio, PacBio data was used for phasing MSDN cluster 3 (see above).

­
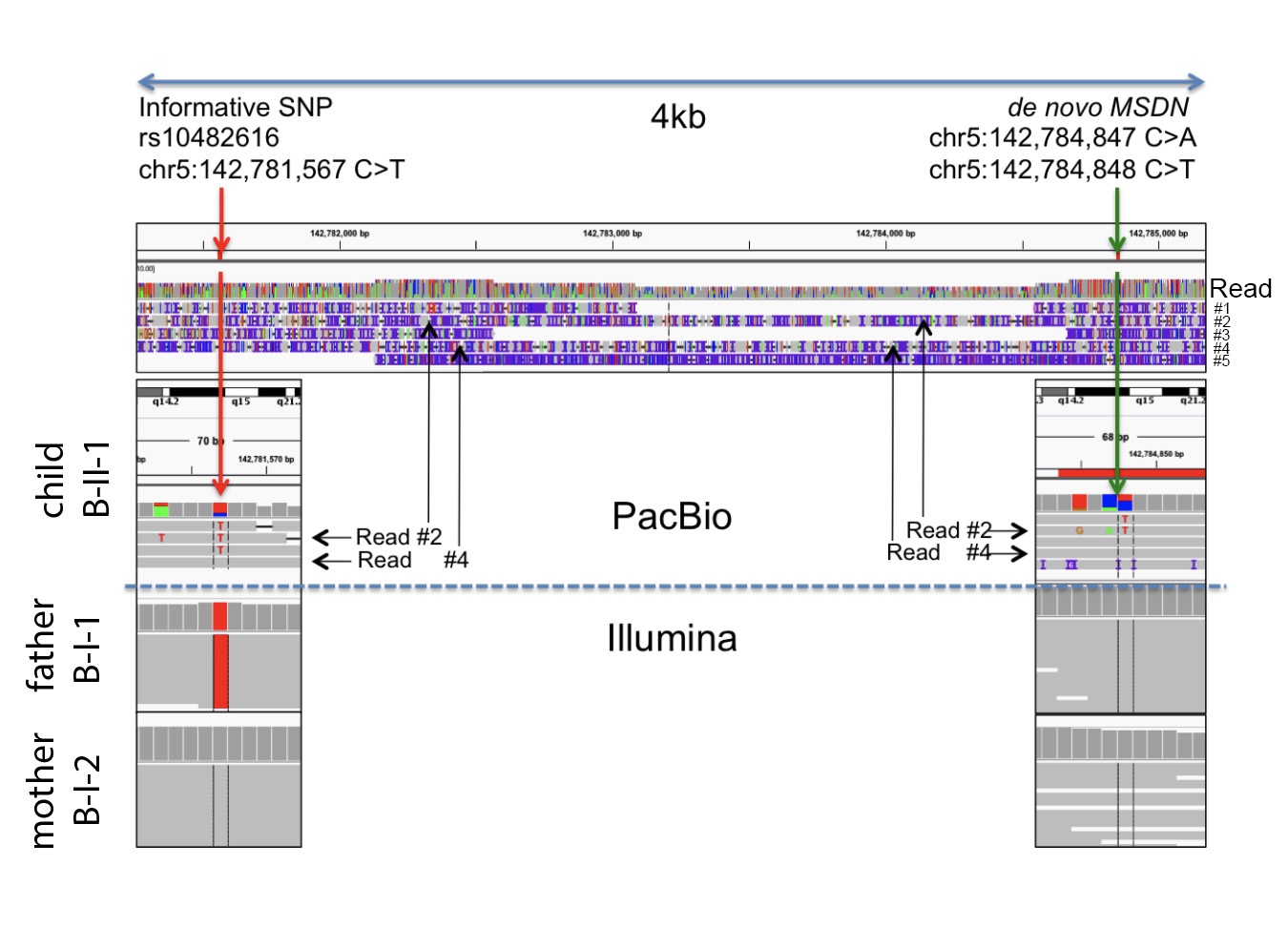


Supplemental Figure 4: The origin of a MSDN can be determined with informative SNPs. In the depicted example the closest informative SNP was found in a distance of about 4kb. Read-backed phasing was possible by long sequences from Pac Bio. Read #2 (second from above) shows SNP rs10482616 that is inherited from the father, B-I-1, and *de novo* MSDN CC>AT. Read #4 does not show the SNP neither the MSDN and originates thus from the maternal chromosome.

*
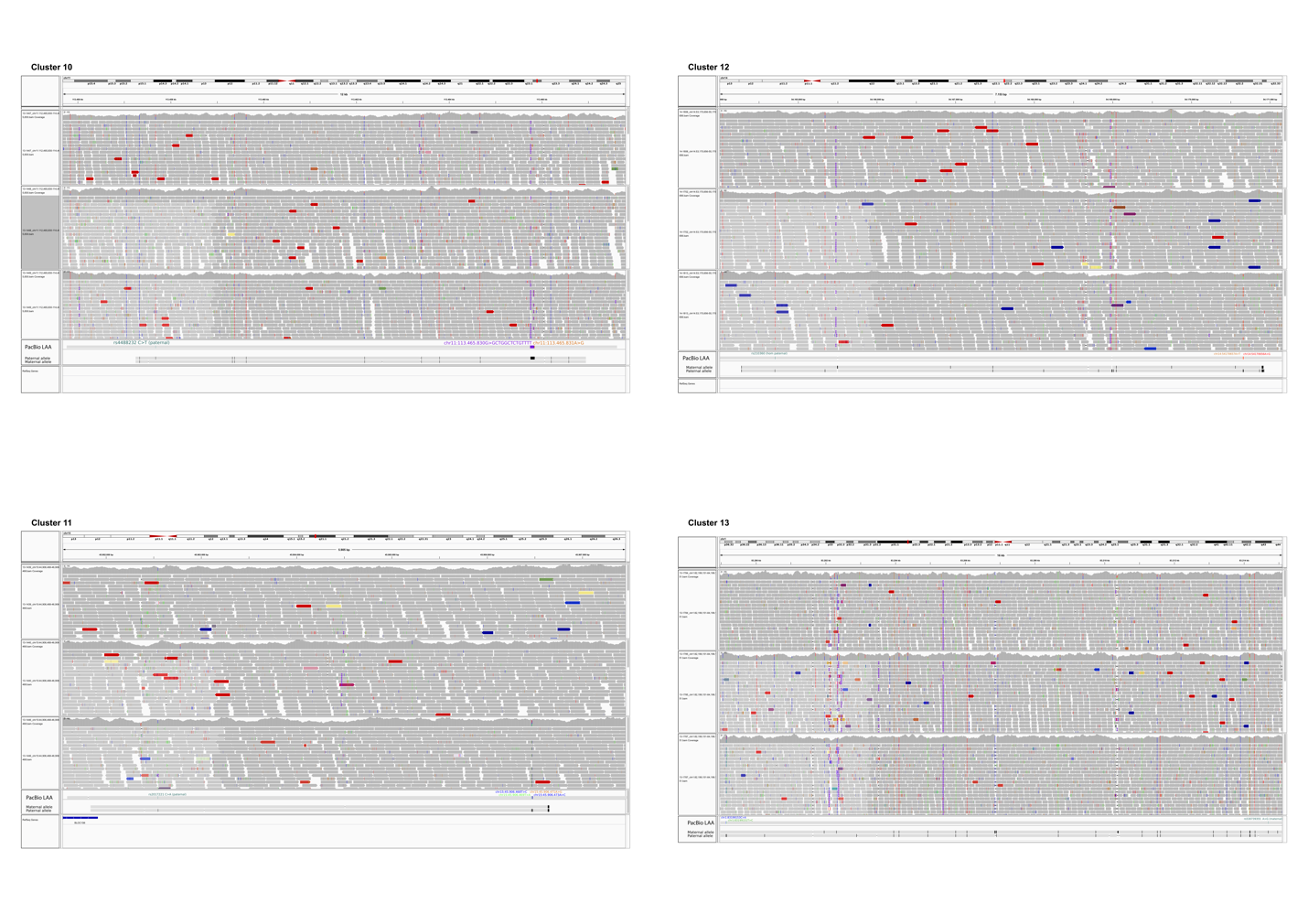
*Supplemental Figure 5: Combination of whole genome short read sequencing on Illumina platform and long read sequencing of PCR amplicons on PacBio platform for phasing of MSDNs. Four clusters (cluster 10, 11, 12, and 13) with MSDNs and a phase informative SNP were amplified by long range PCR, SMARTbell^®^ library preparation was performed and amplicons were sequenced on a PacBio Sequel. Variants were analyzed by Long Amplicon Analysis (LAA) method in Geneious^®^ R11 (Biomatters, New Zealand). Depicted are the short reads from Illumina whole genome sequencing and a consensus sequence of the maternal and paternal allele of PacBio long read sequencing spanning from the phase informative SNP to the MSDN (between 6 and 16kb).

Table S2: Coordinates of all MSDNs with respect to GRCh37 and Sanger validations

| **cluster** | **cohort** | **index** | **ID in Fig1** | **father** | **mother** | **chrom** | **pos** | **ref** | **alt** | **origin** |
| --- | --- | --- | --- | --- | --- | --- | --- | --- | --- | --- |
| 1 | NHL | 16-0048 | A-II-1 | 16-0046 | 16-0047 | chr13 | 73.323.532 | TA | T | paternal |
| 1 | NHL | 16-0048 | A-II-1 | 16-0046 | 16-0047 | chr13 | 73.323.536 | C | T | paternal |
| 1 | NHL | 16-0048 | A-II-1 | 16-0046 | 16-0047 | chr13 | 73.323.541 | T | A | paternal |
| 2 | radar | 13-1452 | B-II-1 | 13-1450 | 13-1451 | chr22 | 19.752.529 | T | C | paternal |
| 2 | radar | 13-1452 | B-II-1 | 13-1450 | 13-1451 | chr22 | 19.752.530 | A | C | paternal |
| 3 | radar | 13-1452 | B-II-1 | 13-1450 | 13-1451 | chr5 | 142.784.847 | C | A | paternal |
| 3 | radar | 13-1452 | B-II-1 | 13-1450 | 13-1451 | chr5 | 142.784.848 | C | T | paternal |
| 4 | radar | 13-1453 | B-II-2 | 13-1450 | 13-1451 | chr19 | 53.870.155 | G | A | paternal |
| 4 | radar | 13-1453 | B-II-2 | 13-1450 | 13-1451 | chr19 | 53.870.156 | G | A | paternal |
| 5 | radar | 13-1453 | B-II-2 | 13-1450 | 13-1451 | chr2 | 157.707.269 | T | G | paternal |
| 5 | radar | 13-1453 | B-II-2 | 13-1450 | 13-1451 | chr2 | 157.707.270 | G | C | paternal |
| **cluster** | **cohort** | **index** | **ID in Fig1** | **father** | **mother** | **chrom** | **pos** | **ref** | **alt** | **origin** |
| 6 | radar | 13-1454 | B-II-3 | 13-1450 | 13-1451 | chr8 | 137.904.684 | T | G | paternal |
| 6 | radar | 13-1454 | B-II-3 | 13-1450 | 13-1451 | chr8 | 137.904.685 | G | C | paternal |
| 7 | radar | 13-1454 | B-II-3 | 13-1450 | 13-1451 | chr9 | 32.825.976 | A | C | paternal |
| 7 | radar | 13-1454 | B-II-3 | 13-1450 | 13-1451 | chr9 | 32.825.977 | C | A | paternal |
| 8 | radar | 13-1455 | C-II-1 | 13-1492 | 13-1493 | chr12 | 78.691.338 | C | T | paternal |
| 8 | radar | 13-1455 | C-II-1 | 13-1492 | 13-1493 | chr12 | 78.691.349 | G | C | paternal |
| 9 | radar | 13-1523 | C-II-2 | 13-1492 | 13-1493 | chr7 | 121.310.969 | A | C | not ascertained |
| 9 | radar | 13-1523 | C-II-2 | 13-1492 | 13-1493 | chr7 | 121.310.970 | G | C | not ascertained |
| 10 | radar | 13-1446 | E-II-2 | 13-1439 | 13-1440 | chr15 | 45.906.468 | T | C | paternal |
| 10 | radar | 13-1446 | E-II-2 | 13-1439 | 13-1440 | chr15 | 45.906.469 | T | A | paternal |
| 10 | radar | 13-1446 | E-II-2 | 13-1439 | 13-1440 | chr15 | 45.906.471 | A | G | paternal |
| 10 | radar | 13-1446 | E-II-2 | 13-1439 | 13-1440 | chr15 | 45.906.473 | A | C | paternal |
| 11 | radar | 13-1449 | D-II-1 | 13-1447 | 13-1448 | chr11 | 113.465.830 | G | GCTGGCTCTGTTTT | paternal |
| 11 | radar | 13-1449 | D-II-1 | 13-1447 | 13-1448 | chr11 | 113.465.831 | A | G | paternal |
| 12 | radar | 13-1787 | F-II-1 | 13-1784 | 13-1785 | chr1 | 83.199.151 | T | C | paternal |
| 12 | radar | 13-1787 | F-II-1 | 13-1784 | 13-1785 | chr1 | 83.199.153 | C | A | paternal |
| 13 | radar | 14-1813 | G-II-3 | 14-1702 | 14-1806 | chr14 | 54.170.656 | A | G | paternal |
| 13 | radar | 14-1813 | G-II-3 | 14-1702 | 14-1806 | chr14 | 54.170.657 | A | T | paternal |

| **cluster** | **cohort** | **index** | **ID in Fig1** | **father** | **mother** | **chrom** | **pos** | **ref** | **alt** | **origin** |
| --- | --- | --- | --- | --- | --- | --- | --- | --- | --- | --- |
| 14 | control | 3590 |  | 3591 | 3592 | chr3 | 135.570.694 | A | G | not ascertained |
| 14 | control | 3590 |  | 3591 | 3592 | chr3 | 135.570.695 | A | T | not ascertained |
| 15 | control | 4838 |  | 4839 | 4840 | chr10 | 25.241.432 | C | T | not ascertained |
| 15 | control | 4838 |  | 4839 | 4840 | chr10 | 25.241.433 | C | T | not ascertained |
| 16 | control | R14-225 |  | R14-230 | R14-229 | chr11 | 98.054.812 | T | C | paternal |
| 16 | control | R14-225 |  | R14-230 | R14-229 | chr11 | 98.054.815 | C | A | paternal |
| 17 | control | R14-225 |  | R14-230 | R14-229 | chr9 | 126.531.330 | T | G | paternal |
| 17 | control | R14-225 |  | R14-230 | R14-229 | chr9 | 126.531.347 | T | C | paternal |
| 18 | control | R14-235 |  | R14-237 | R14-236 | chr4 | 15.046.856 | G | A | paternal |
| 18 | control | R14-235 |  | R14-237 | R14-236 | chr4 | 15.046.862 | G | A | paternal |


 Supplemental Figure 6: Sanger sequencing chromatograms of MSDNs in trios.

**Parental origin of translocations**


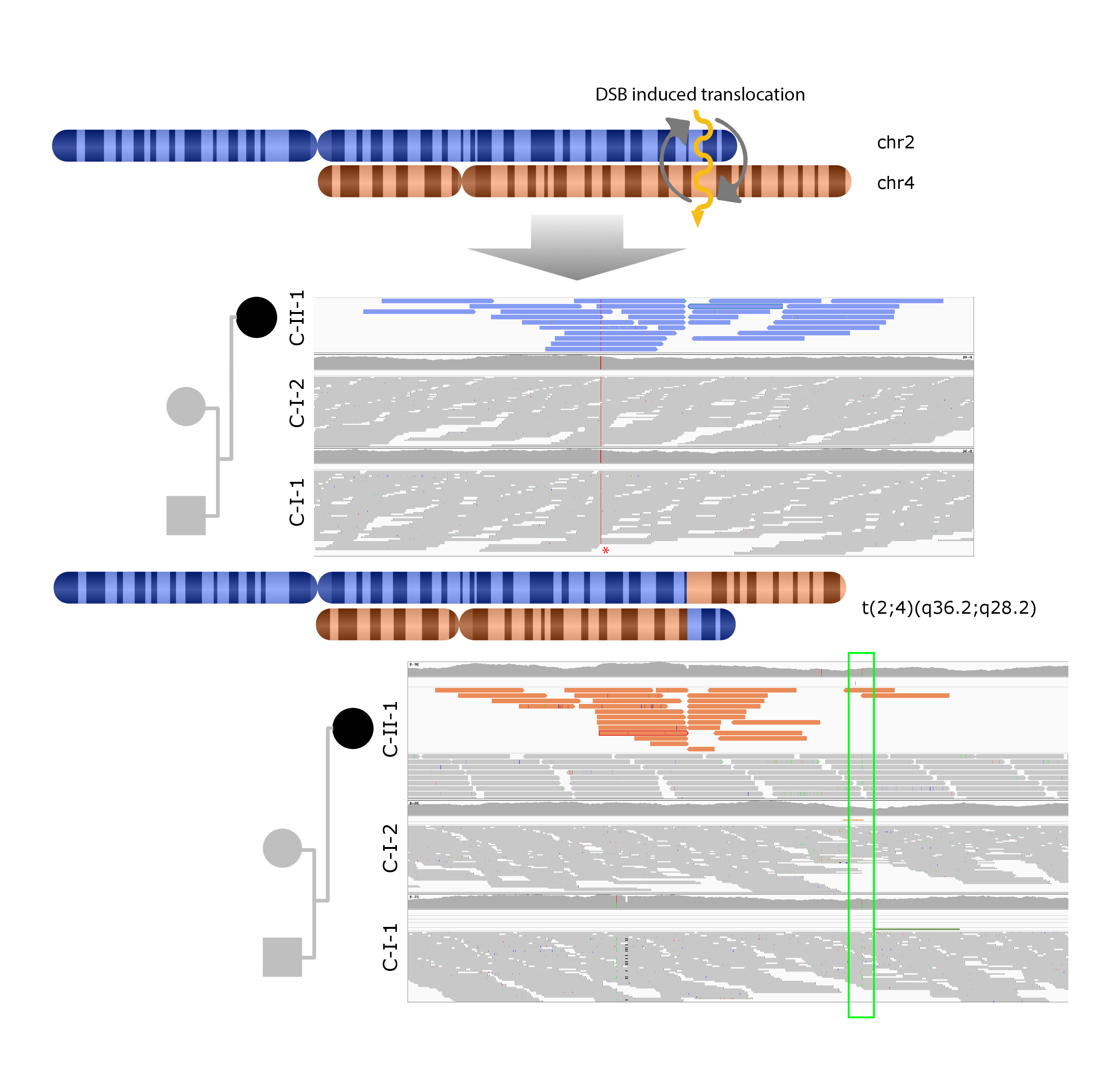


Supplemental Figure 7: The parental origin of the translocation could be determined with reads pairs that span the breakpoint and cover an informative SNP (green box). The first read that maps to chromosome 2 is colored in blue, whereas the second read of the pair that maps to chromosome 4 is colored in brown. The read depicted in the green box, shows a SNP that is inherited from the paternal side, indicating the origin of the de novo translocation in the father’s germline. The asterisk indicates a SNP that is homozygous in all individuals.


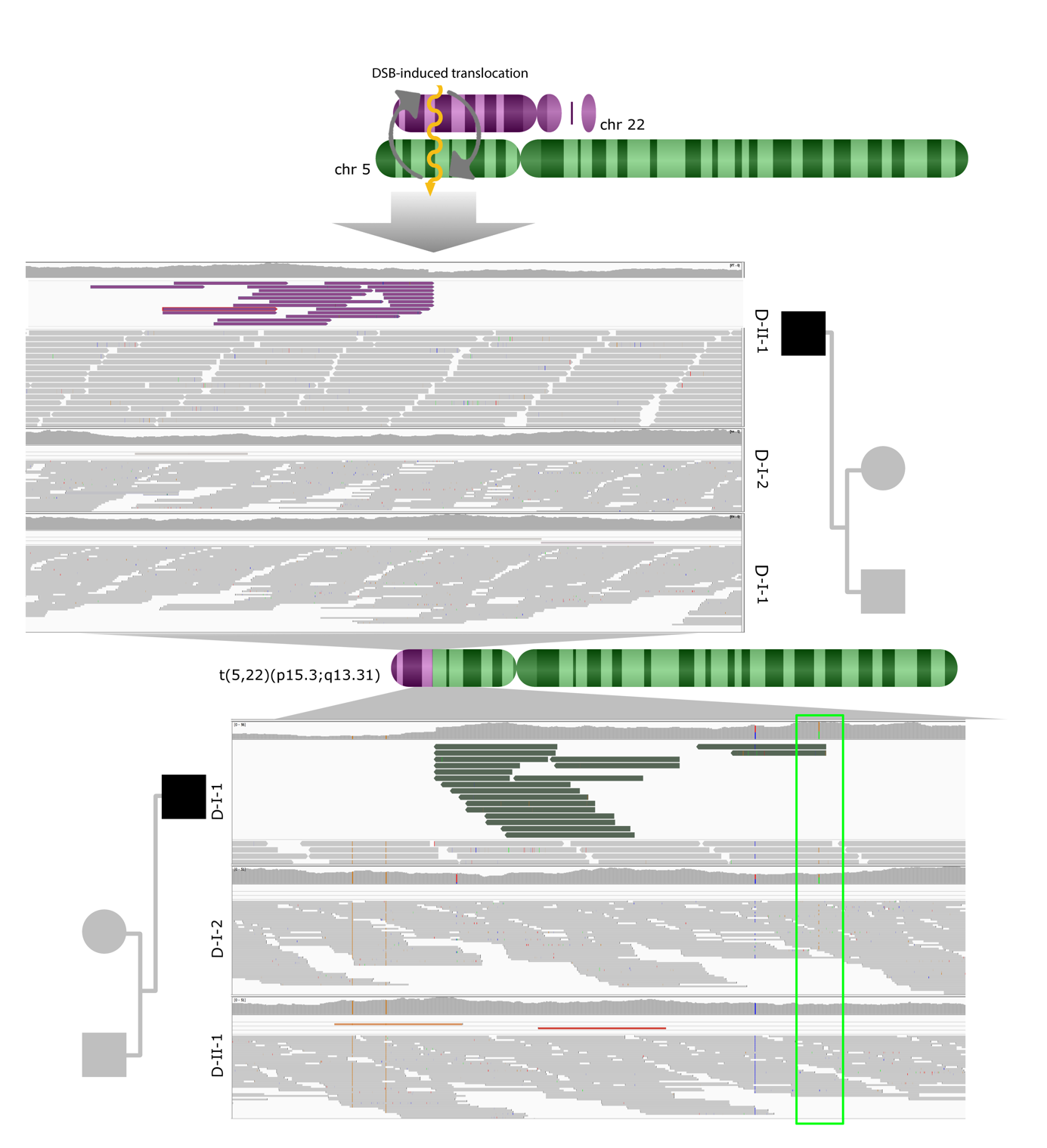


Supplemental Figure 8: The parental origin of the translocation could be determined with read pairs that span the breakpoint and cover an informative SNP (green box). The end of the sequenced DNA fragment that maps to chromosome 22 is colored in violet, whereas the other end that maps to chromosome 5 is colored in dark green. The green box frames a position that is heterozygous in the mother and offspring. Two reads spanning the breakpoint show the wildtype allele, that was inherited from the paternal site. Note, as D-I-1 has a 5p deletion there are no read pairs from the telomeric site.
